# Retraction: detection and differentiation of *borrelia burgdorferi *sensu lato in ticks collected from sheep and cattle in China

**DOI:** 10.1186/1746-6148-7-56

**Published:** 2011-09-20

**Authors:** Qingli Niu, Guiquan Guan, Jifei Yang, Yuguang Fu, Zongke Xu, Youquan Li, Miling Ma, Zhijie Liu, Junlong Liu, Aihong Liu, Qiaoyun Ren, Wayne Jorgensen, Jianxun Luo, Hong Yin

**Affiliations:** 1 State Key Laboratory of Veterinary Etiological Biology, Key Laboratory of Veterinary Parasitology of Gansu Province, Key Laboratory of Grazing Animal Diseases MOA, Lanzhou Veterinary Research Institute, Chinese Academy of Agricultural Sciences, Lanzhou 730046, China; 2 Centre of Animal Science, QAAFI, University of Queensland 4072 Australia

## Retraction

This article [1] has been regretfully retracted due to plagiarism by the authors. The method for the identification of *Borrelia burgdorferi sensu lato *species in ticks presented in the article was originally developed and published by Sjoerd Rijpkema and colleagues [2]. The authors apologise to all affected parties for the inconvenience caused.
